# Intraocular Pressure-Lowering Potential of Subthreshold Selective Laser Trabeculoplasty in Patients with Primary Open-Angle Glaucoma

**DOI:** 10.1155/2016/2153723

**Published:** 2016-07-27

**Authors:** Hong Yang Zhang, Yong Jie Qin, Yang Fan Yang, Jian Gang Xu, Min Bin Yu

**Affiliations:** ^1^Department of Ophthalmology, Guangdong Eye Institute, Guangdong General Hospital and Guangdong Academy of Medical Sciences, Guangzhou 510080, China; ^2^Zhongshan Ophthalmic Center, State Key Laboratory of Ophthalmology, Sun Yat-sen University, Guangzhou 510060, China

## Abstract

*Purpose. *To compare the efficacy of subthreshold and conventional selective laser trabeculoplasty (SLT) in lowering intraocular pressure (IOP) in the patients with primary open-angle glaucoma (POAG).* Methods. *Fifty-two eyes from fifty-two POAG patients were randomized into two groups, one group treated with subthreshold SLT using two-thirds of the conventional energy and the other one treated with the conventional energy. IOP was measured with the Goldmann tonometer and the anterior chamber inflammation was determined using laser flare meter.* Results. *The initial energy dosage used in subthreshold SLT group was significantly lower than the amount of the energy used in conventional SLT group (0.4 ± 0.1 mJ versus 0.6 ± 0.1 mJ, *P* = 0.030). The total energy dosage was also significantly lower in subthreshold SLT group compared to the other group (37.6 ± 3.3 mJ versus 51.8 ± 5.7 mJ, *P* = 0.036). However, the level of inflammation in aqueous humor, amount of reduction in IOP, and the success rate in controlling IOP was the same in both groups.* Conclusion. *The efficacy of subthreshold SLT group in reducing IOP in POAG patients is comparable to the efficacy of conventional SLT group.

## 1. Background

Selective laser trabeculoplasty (SLT) was considered as an effective and safe surgery for lowering intraocular pressure (IOP) [1–3] since it has initially been described in 1995 [4]. SLT, a 532 nm frequency-doubled and Q-switched neodymium (Nd): yttrium-aluminum-garnet (YAG) laser, is capable of targeting pigmented trabecular cells without producing thermal damage to the adjacent nonpigmented cells or structures of trabecular meshwork [1, 4].* In vitro* histological observations of the human trabecular meshwork demonstrated that cracking of the intracytoplasmic pigment granules and disruption of the trabecular endothelial cells were the only findings after SLT. In addition, these studies showed that coagulative damage or disruption of the corneoscleral or uveal trabecular beam structure never happens after SLT [5]. In some recent studies that compared latanoprost and SLT in reducing IOP, both approaches had the similar efficacy, but SLT was more cost effective [6, 7]. In fact, in medication group, 27% of the patients required additional treatments due to the failure of IOP control whereas 11% of patients in SLT group needed more treatment sessions [7, 8]. Recently, SLT has been recommended as the first-line treatment for the patients with open-angle glaucoma, including primary open-angle glaucoma (POAG), pigmentary glaucoma, and exfoliative glaucoma [9–11].

SLT does not cause a substantial decrease in IOP (only 5-6 mmHg) [12] and the long-term follow-up of these patients revealed that further interventions were needed [2]. As SLT does not cause any scaring of trabecular meshwork, it could be repeated multiple times [13]. A retrospective study showed that a new session of SLT could cause a significant reduction in IOP in 24 months when an initially successful SLT fails over time [14]. In one study that compared SLT with conventional argon laser trabeculoplasty (ALT), the energy released during treatment and the amount of immediate postoperative inflammation in the anterior chamber were significantly lower in SLT. In comparison with conventional trabeculoplasty with argon, SLT was better tolerated; and the level of discomfort during treatment was much lesser [15]. It is preferred to repeat SLT in order to reduce or eliminate the need for medication in the patients with open-angle glaucoma [16]. The needed amount of the energy for conventional SLT is 0.1 megajoule (mJ) less than the amount of the energy that generates “champagne-like bubbles” on the surface of the trabecular meshwork, although it has been shown that IOP-lowering effect of SLT can be achieved even if “champagne-like bubbles” have not been generated. Thus, if lower energy SLT could be as effective as a conventional SLT, it would be an advantage for the patients who require multiple SLT interventions. In a study, the IOP-lowering effect of the half-energy SLT in patients with ocular hypertension or POAG was comparable to the conventional SLT and the complications in half energy dosage group, for example, mild pain, conjunctival hyperemia, and transient IOP spike, were much lesser [17]. The optimum dosage for reduced energy SLT, however, needs further investigation. SLT-induced inflammation has an important role in modulating the extracellular matrix profile to clean the trabecular meshwork and facilitating outflow of the aqueous humor [18]. Thus, the present study was conducted to apply lower level of laser energy for SLT and evaluate its effect on generating anterior chamber inflammation to lower IOP in POAG patients.

## 2. Methods

In this prospective observational case series, fifty-two eyes of fifty-two patients with POAG were included. Demographics of these patients were summarized in Table 1. To be considered as POAG, patients should have glaucomatous optic nerve head or nerve fiber abnormalities, with or without visual field defects. That was based on the criteria of the International Society of Geographical and Epidemiological Ophthalmology* (ISGEO)*. This study was approved by the ethics committee of Zhongshan Ophthalmic Center, Sun Yat-sen University. All patients signed informed consent forms. Patients were followed up for 12 months. Eligible patients for the study were the POAG patients with IOP higher than 21 mmHg. Most of the patients included were treated with SLT as primary treatment, and those who were taking antiglaucoma medications underwent one-month washout before SLT. Exclusion criteria were as follows: (i) secondary open-angle glaucoma; (ii) a cup to disc ratio larger than 0.9; (iii) remaining of only 5°–10° of central visual field or having only a preserved temporal island; (iv) one-eye patients; (v) patients on topical or systemic corticosteroid.

The 360-degree SLT treatment was performed on the entire meshwork with the Nd: YAG laser system (Ellex Medical Pty. Ltd., Adelaide, Australia) with 532 nm wavelengths, 3-nanosecond (ns) pulse width, 400 *μ*m spot size, and 0.3 to 2.6 mJ energy. The initial energy dosage was set at 0.8 mJ and adjusted by 0.1 mJ each time until the champagne-like bubbles could be appreciated on the surface of the trabecular meshwork (threshold energy). The conventional energy for treatment is 0.1 mJ lower than the threshold energy. We defined the subthreshold energy as two-thirds of the conventional energy. Pigmented trabecular meshwork was treated in 100 points—25 in each quadrant without overlapping—in 360°. The treatment energy and the total energy were recorded accordingly.

Fifty-two Participants were randomly divided into two groups with equal numbers. One group was treated with conventional SLT and another treated with subthreshold SLT. Surgeries were performed under topical anesthesia after the assessment of trabecular meshwork pigmentation with gonioscopy according to the Scheie system (0 = no pigmentation, to 4 = dense pigmentation). None of the patients were treated with topical steroids, nonsteroidal anti-inflammatory drugs, and antiglaucoma medications after SLT. IOP was measured by the Goldmann tonometer (mounted on slit lamps, AT 900®Haag-Streit Inc., USA) during one-year follow-up time. Laser flare cell meter (FC-2000, Kowa Company Limited, Japan) was used to determine the anterior chamber inflammation amount including the total protein contents and the cell density two hours, one day, seven days, and one month after SLT. The average IOP was determined by the mean value of three separate measurements at 10:00 ± 0.5 h am. Total protein content and cell density in the anterior chamber were determined based on the mean of five separate sessions of measurement for every eye. Best corrected visual acuity (BCVA) was measured using standard distant visual acuity chart. Surgery was considered successful when a postoperational decrease in IOP greater than or equal to 20% of pretreatment read was encountered, provided that patient had not received any additional treatment (neither medical nor surgical) [10, 19].

Most data were analyzed using the nonparametric Kruskal-Wallis test. Comparisons between two groups were done by the Mann-Whitney test. Chi-square test was used to analyze categorical data. Significance was set at *P* < 0.05 for the all tests. Statistical analyses were performed using SPSS statistical software package (version 20.0).

## 3. Results

The pretreatment demographic characteristics of the conventional and subthreshold SLT groups were shown in Table 1. There was no statistically significant difference in age, IOP, visual acuity, refraction, BCVA, central corneal thickness (CCT), cup to disc ratio (*C*/*D*), and the mean grade of trabecular meshwork pigmentation between the two groups.

The initial energy dosages used in subthreshold SLT group and the conventional one were 0.4 ± 0.1 mJ and 0.6 ± 0.1 mJ, respectively. These amounts were significantly different (*P* = 0.030). The total energy dosage for SLT treatment was 37.6 ± 3.3 mJ in subthreshold group and 51.8 ± 5.7 mJ in conventional group, which were also significantly different (*P* = 0.036). The data were shown in Table 2.

The amount of decrease in IOP after conventional group and subthreshold SLT group is presented in Table 3. The mean IOP before conventional SLT group and subthreshold SLT group was 25.0 ± 2.5 mmHg and 25.7 ± 1.9 mmHg, respectively, that were not significantly different (*P* = 0.059). One day after intervention, IOP was significantly decreased (*P* < 0.05) compared to its pretreatment amount. The amount of IOP was stable up to 12 months after surgery. The highest amount of decrease was found on the first day after SLT treatment, in which there were 26.0% reduction in conventional group and 27.2% reduction in subthreshold group (*P* = 0.02, *P* = 0.07, resp.).

Analysis of the anterior chamber inflammation using laser flare cell meter revealed that, 2 hours after treatment, the concentration of protein increased from 3.4 ± 1.0 pc/ms to 9.1 ± 5.6 pc/ms in conventional SLT group and from 3.7 ± 1.0 pc/ms to 9.6 ± 6.7 pc/ms in subthreshold SLT group, respectively (*P* < 0.001). There was not any statistically significant difference in total protein concentration (Table 4) and cell density of aqueous humor (Table 5) between conventional SLT group and subthreshold SLT group in month one visit. Protein concentration gradually decreased in day one, day seven, and month one visits (Table 4). A similar trend in inflammatory response was found in the cell density of aqueous humor (Table 5). Maximum infiltration of the cells into aqueous humor (11.1 ± 10.05 cells/0.5 m^3^ in conventional SLT group and 11.4 ± 9.9 cells/0.5 m^3^ in subthreshold SLT group) happened 2 hours after SLT treatment. The number of infiltrating cells decreased to 0.4 ± 0.5 cells/0.5 m^3^ in conventional SLT group and to 0.3 ± 0.3 cells/0.5 m^3^ in subthreshold SLT group seven days after intervention.

One day after treatment, 92.3% of patients in the conventional SLT group and 96.2% patients in the subthreshold SLT group met the criteria for successful treatment. After one year, successful control of IOP in over half of the treated eyes was achieved. Although the amount of decrease in IOP was a little bit higher in subthreshold group in comparison with conventional group, this trend was not statistically significant (Table 6).

## 4. Discussion and Conclusion

In this prospective randomized study, we compared the IOP-lowering efficacy of subthreshold SLT with conventional SLT. In addition, we compared their effect in inducing anterior chamber inflammation in the POAG patients. We observed that, immediately after subthreshold SLT, patients' IOP decreased and remained in this reduced range until one year. Also, the success rate of subthreshold SLT was comparable to the conventional SLT. In addition, subthreshold SLT induced inflammation of the anterior chamber in 2 hours that recovered in 24 hours. The amount of inflammation when measured with laser flare cell meter was similar to the amount of the inflammation induced by conventional SLT.

In a previous study, the dosage of the energy used in SLT was positively correlated with the amount of IOP reduction [20]. A meta-analysis showed that there was no statistically significant difference between SLT and ALT in terms of the amount of reduction of IOP [21, 22]. The impact of the perimeter of the angel that laser therapy is applied on (180-degree SLT versus 360-degree SLT) in lowering IOP is negligible [19]. On the other hand, if the number of laser spots increases from 25 to 50 in every quadrant the efficacy of SLT in lowering IOP will decrease [23]. Although it is not clear whether increasing energy dosage could enhance the effect of SLT or not, it is generally accepted that adjustment of the energy level per spot to the lowest possible amount is crucial in obtaining the highest possible efficacy of SLT. In the present study we observed that subthreshold SLT with two-thirds of the conventional energy has enough efficacy in lowering IOP in POAG patients. In some patients repeated sessions of conventional SLT is needed to achieve a long-lasting IOP control [14, 24, 25]; thus a reduced energy intervention would be more appropriate in these patients.

Our study confirmed a previous report about the comparability of IOP-lowering efficacy of half-dose SLT with the conventional SLT [17]. There was not any statistically significant difference in success rate of the reduced energy SLT compared to the conventional approach and its efficacy in lowering IOP was almost equal. The IOP-lowering effect of SLT is likely related to the inflammatory mediators [26]. We observed that subthreshold and conventional SLT both induce the same amount of anterior chamber inflammation that can explain their similar efficacy in controlling IOP. An intensive increase in anterior chamber inflammation (increase in total protein contents and infiltration of inflammatory cells in aqueous humor) only two hours after SLT could be the reason for IOP reduction one day after surgery. However, this inflammation could end up with corneal edema and full-blown anterior uveitis. Thus, anti-inflammatory medications are needed after SLT treatment [11, 27, 28]. These therapies do not have any influence on the therapeutic effect of SLT [29]. It is not clear what degree of inflammation in anterior chamber following SLT is beneficial in IOP control. As our patients did not need any anti-inflammatory medication, application of a reduced energy SLT might be a safer way for controlling IOP.

The pigmentation at the trabecular meshwork was considered to be related to the pressure-lowering effect of SLT [30], but no correlation was found in our patients in one-year follow-up, which agrees with other studies [8, 27]. So the better response to SLT may not be explained solely with the degree of trabecular meshwork pigmentation. The exact mechanism of SLT is still unclear, and studies found that applying laser on the trabecular meshwork cells can result in the secretions of some cytokines, for example, interleukin-1 alpha/beta (IL-1*α*/*β*) and tumor necrosis factor-alpha (TNF-*α*), which activates macrophages and upregulates expression of matrix metalloproteinases (MMPs) leading to the reconstitution of trabecular meshwork [28, 31, 32]. The number of monocytes in human trabecular meshwork or monkey eyes increases 4- to 5-fold following SLT treatment [33]. SLT-induced MMPs expression, for example, MMP-3 and MMP-9, was known to be mediated by IL-1*β* and TNF-*α* through activation of c-Jun N-terminal kinase in trabecular meshwork [34, 35]. These molecules are considered very important in facilitating the outflow of aqueous humor [36]. It was believed that a low-grade inflammation could be induced by laser therapy of the rabbit eyes via spreading the cytokines to aqueous humor. Concentration of the inflammatory mediators returns to the normal level 3–7 days after SLT without any medication [37]. In human eyes, a mild inflammation is also induced with 180-degree SLT. This inflammation is detectable one hour after intervention and is completely resolved within five days [1]. In our study very similar to the previous reports total protein exudation and cellular infiltration were in their highest level two hours after SLT and cleared on seventh day after intervention. It indicates that after subthreshold SLT only a low-grade immune response happens, which is not the case with conventional SLT although their IOP-lowering efficacy is the same.

This study has some limitations: first, the sample size is small. Second, observation time is short. Long-term follow-up is needed to further compare the conventional and reduced dose SLT.

In summary, this study provides a guideline for adjusting the minimum amount of energy needed for SLT. In comparison with conventional dose, the same therapeutic effect could be achieved with less energy.

## Figures and Tables

**Table 1:** Pretreatment patient characteristics.

	Conventional SLT group	Subthreshold SLT group	*P *value^*∗*^
Age (year)	42.9 ± 14.3	46.8 ± 15.2	0.976
IOP (mmHg)	25.0 ± 2.5	25.7 ± 1.9	0.059
CCT (*μ*m)	540.3 ± 24.0	544.0 ± 27.1	0.569
Refraction (D)	−3.2 ± 2.7	−2.8 ± 2.3	0.147
*C*/*D*	0.7 ± 0.1	0.7 ± 0.1	0.940
BCVA	1.0 ± 0.2	1.1 ± 0.3	0.750
TM pigmentation	1.93 ± 0.78	1.79 ± 0.92	0.144

Data shown were presented as mean ± SD, Mann-Whitney test, *n* = 26. ^*∗*^ *P* value: subthreshold SLT group versus conventional SLT group. IOP: intraocular pressure; CCT: central corneal thickness; D: diopter; *C*/*D*: the cup to disc ratio; BCVA: best corrected visual acuity; TM: trabecular meshwork; SD: standard deviation.

**Table 2:** Energy dosage (mJ) used in conventional SLT group and subthreshold SLT group.

Group	Conventional SLT group	Subthreshold SLT group	*P *value^*∗*^
Initial energy	0.6 ± 0.1	0.4 ± 0.1	0.030
Total energy	51.8 ± 5.7	37.6 ± 3.3	0.036

Data shown were presented as mean ± SD, Mann-Whitney test, *n* = 26. ^*∗*^ *P* value: subthreshold SLT group versus conventional SLT group.

**Table 3:** Intraocular pressure (IOP, mmHg) in conventional SLT group and subthreshold SLT group.

Time	Conventional SLT group	Subthreshold SLT group	*P* value^*∗*^
Pretreatment	25.0 ± 2.5	25.7 ± 1.9	0.059
2 hours	21.0 ± 2.2 (0.441)^†^	22.4 ± 2.2 (0.092)^†^	0.713
1 day	18.5 ± 1.9 (0.020)^†^	18.7 ± 2.1 (0.070)^†^	0.597
7 days	19.9 ± 1.7 (0.010)^†^	19.9 ± 1.8 (0.030)^†^	0.169
1 month	19.7 ± 2.0 (0.020)^†^	19.6 ± 1.9 (0.018)^†^	0.581
3 months	19.4 ± 2.1 (0.020)^†^	19.5 ± 1.8 (0.020)^†^	0.433
6 months	19.7 ± 1.8 (0.000)^†^	19.5 ± 1.9 (0.002)^†^	0.204
12 months	20.0 ± 1.7 (0.008)^†^	20.3 ± 1.6 (0.020)^†^	0.076

Data shown were presented as mean ± SD, Mann-Whitney test, *n* = 26. ^*∗*^ *P* value: subthreshold SLT group versus conventional SLT group. ^†^ *P* value: IOP at each follow-up visit comparing to pretreatment baseline.

**Table 4:** Protein concentration of aqueous humor in conventional SLT group and subthreshold SLT group.

Time	Conventional SLT group	Subthreshold SLT group	*P* value^*∗*^
Pretreatment	3.4 ± 1.0	3.7 ± 1.0	0.640
2 hours	9.1 ± 5.6 (0.000)^†^	9.6 ± 6.7 (0.000)^†^	0.545
1 day	4.1 ± 1.6 (0.002)^†^	3.9 ± 2.2 (0.160)^†^	0.863
7 days	3.1 ± 1.4 (0.169)^†^	3.5 ± 1.2 (0.479)^†^	0.674
1 month	3.5 ± 1.4 (0.137)^†^	3.3 ± 1.4 (0.413)^†^	0.799

Data shown were presented as mean ± SD, Mann-Whitney test, *n* = 26. ^*∗*^ *P* value: subthreshold SLT group versus conventional SLT group. ^†^ *P* value: protein concentration of aqueous humor at each follow-up visit comparing to the pretreatment baseline.

**Table 5:** Cell density of aqueous humor in conventional SLT group and subthreshold SLT group.

Time	Conventional SLT group	Subthreshold SLT group	*P* value^*∗*^
Pretreatment	0.3 ± 0.2	0.3 ± 0.2	0.112
2 hours	11.1 ± 10.5 (0.000)^†^	11.4 ± 9.9 (0.000)^†^	0.527
1 day	1.3 ± 1.3 (0.000)^†^	1.1 ± 1.2 (0.001)^†^	0.879
7 days	0.4 ± 0.5 (0.04)^†^	0.3 ± 0.3 (0.046)^†^	0.351
1 month	0.3 ± 0.4 (0.178)^†^	0.3 ± 0.3 (0.098)^†^	0.829

Data shown were presented as mean ± SD, Mann-Whitney test, *n* = 26. ^*∗*^ *P* value: subthreshold SLT group versus conventional SLT group. ^†^ *P* value: cell density of aqueous humor at each follow-up visit comparing to the pretreatment baseline.

**Table 6:** Successful cases that reach ≥20% decrease in IOP after SLT treatment.

Group	2 hours	1 day	7 days	1 month	3 months	6 months	12 months
Conventional SLT group	7/26 (26.9%)	24/26 (92.3%)	18/26 (69.2%)	18/26 (69.2%)	17/26 (65.4%)	18/26 (69.2%)	15/21 (71.4%)
Subthreshold SLT group	6/26 (23.1%)	25/26 (96.2%)	20/26 (76.9%)	20/26 (76.9%)	18/26 (69.2%)	19/26 (73.1%)	16/21 (76.2%)
*P* value^*∗*^	0.749	0.552	0.532	0.532	0.532	0.760	1.00

Data shown were presented as the number of successful cases over total number of POAG patients, Chi-square test. ^*∗*^ *P* value: subthreshold SLT group versus conventional SLT group in each follow-up visit.
